# Accuracy and Trending Ability of Electrical Biosensing Technology for Non-invasive Cardiac Output Monitoring in Neonates: A Systematic Qualitative Review

**DOI:** 10.3389/fped.2022.851850

**Published:** 2022-03-17

**Authors:** Lizelle Van Wyk, Samir Gupta, John Lawrenson, Willem-Pieter de Boode

**Affiliations:** ^1^Division Neonatology, Department of Pediatrics and Child Health, Stellenbosch University and Tygerberg Children’s Hospital, Cape Town, South Africa; ^2^Department of Engineering and Medical Physics, Durham University, Durham, United Kingdom; ^3^Division of Neonatology, Sidra Medicine, Doha, Qatar; ^4^Pediatric Cardiology Unit, Department of Pediatrics and Child Health, Stellenbosch University, Cape Town, South Africa; ^5^Division of Neonatology, Department of Perinatology, Radboud University Medical Center, Radboud Institute for Health Sciences, Amalia Children’s Hospital, Nijmegen, Netherlands

**Keywords:** systematic review, bioimpedance, bioreactance, neonates, trending ability, accuracy and bias

## Abstract

**Background:**

Electrical biosensing technology (EBT) is an umbrella term for non-invasive technology utilizing the body’s fluctuating resistance to electrical current flow to estimate cardiac output. Monitoring cardiac output in neonates may allow for timely recognition of hemodynamic compromise and allow for prompt therapy, thereby mitigating adverse outcomes. For a new technology to be safely used in the clinical environment for therapeutic decisions, it must be proven to be accurate, precise and be able to track temporal changes. The aim of this systematic review was to identify and analyze studies that describe the accuracy, precision, and trending ability of EBT to non-invasively monitor Left ventricular cardiac output and/or stroke volume in neonates.

**Methods:**

A qualitative systematic review was performed. Studies were identified from PubMed NCBI, SCOPUS, and EBSCOHost up to November 2021, where EBT technologies were analyzed in neonates, in comparison to a reference technology. Outcome measures were bias, limits of agreement, percentage error for agreement studies and data from 4-quadrant and polar plots for trending studies. Effect direction plots were used to present results.

**Results:**

Fifteen neonatal studies were identified, 14 for agreement and 1 for trending analysis. Only thoracic electrical biosensing technology (TEBT), with transthoracic echocardiography (TTE) as the comparator, studies were available for analyzes. High heterogeneity existed between studies. An equal number of studies showed over- and underestimation of left ventricular output parameters. All studies showed small bias, wide limits of agreement, with most studies having a percentage error >30%. Sub-analyses for respiratory support mode, cardiac anomalies and type of technology showed similar results. The single trending study showed poor concordance, high angular bias, and poor angular concordance.

**Discussion:**

Overall, TEBT shows reasonable accuracy, poor precision, and non-interchangeability with TTE. However, high heterogeneity hampered proper analysis. TEBT should be used with caution in the neonatal population for monitoring and determining therapeutic interventions. The use of TEBT trend monitoring has not been sufficiently studied and requires further evaluation in future trials.

## Introduction

Adequate systemic perfusion is dependent on cardiac output (CO), as determined by heart rate (HR) and stroke volume (SV) and influenced by systemic vascular resistance (SVR). A complex interaction exists between HR, blood flow, SVR and blood pressure (BP) to ensure that cellular metabolic oxygen demand is met (1). The circulatory system of neonates is significantly different from that of adults or children, as the neonatal population is a heterogeneous mix of gestational and postconceptional ages, with different degrees of cardiovascular maturation (2). Indirect measures of CO, such as HR and BP, are inadequate for the assessment of neonatal hemodynamic status (3).

CO is considered a fundamental physiological parameter for diagnosis and guidance of therapy in various neonatal conditions (4). Maintaining optimal perfusion and oxygenation is of prime concern in neonatal intensive care units (NICU). Comprehensive monitoring of various physiological variables is required, as low CO has been associated with increased morbidity, adverse neurodevelopmental outcome, and increased mortality (5).

CO measurement, *via* invasive techniques (e.g., intermittent pulmonary artery thermodilution and Fick’s method) are considered the gold standards for accurately determining CO (6). However, in neonates these methods are inappropriate (7) as catheters are often too big and the invasiveness of these methods have been questioned, also in adult medicine (8). Minimally invasive cardiac output monitoring technologies encompass devices not requiring the insertion of a pulmonary artery catheter, e.g., pulse contour, pulse power analysis, partial gas re-breathing and transpulmonary ultrasound dilution (9). Some of these technologies require the placement of an arterial line (pulse contour and pulse power analysis) and may need placement of a central venous line for calibration purposes (7). These technologies have been poorly studied in the neonatal population whilst others are still under development (transpulmonary ultrasound dilution) (10). Most other CO measurement technologies in neonates offer only intermittent measurement values as they are labor, skill or technology intensive [transthoracic echocardiography (TTE) and cardiac magnetic resonance imaging (cMRI)] (7).

Non-invasive cardiac output monitoring technologies were therefore developed, offering fully non-invasive methods of monitoring SV and CO. These included intermittent measurements *via* Doppler ultrasound [Ultrasound Cardiac Output Monitor (USCOM) and transthoracic echocardiography (TTE)] and continuous measurements *via* various electrical biosensing technologies (EBT) [bioimpedance (BI) and bioreactance (BR)].

For a new technology to be safely used in the clinical environment, and to allow therapeutic decisions to be based upon it, it must be proven to be accurate and precise. A good agreement between a new technology and a gold standard reference technology is defined by a small bias (indicating a high accuracy), narrow limits of agreement (indicating a high precision) and a percentage error ≤30% (indicating technology interchangeability) (11, 12). Trending ability (change over time) should also be assessed to ensure that the new technology’s direction and magnitude of change is in line with that of the reference technology (13).

## Technology Background

The first type of non-invasive cardiac monitoring, rheocardiography, was developed in 1949 by Kedrov (14) but only found popularity in 1966 when Kubicek re-designed it for use in the aerospace industry (15). Since then, numerous iterations of this technology have become available in the healthcare industry, with methodologies measuring changes in whole body, segmental or thoracic impedance from which stroke volume (SV) and hence cardiac output (CO) are derived. Numerous nomenclatures are used – whole body electrical bioimpedance (WBEB), thoracic electrical bioimpedance (TEB), electrical velocimetry, electrical cardiometry, impedance cardiometry, impedance cardiography, thoracocardiography, bioreactance, and rheocardiography. These have subtle differences, often with proprietary algorithms and models to estimate SV and CO.

For EBT a high frequency, low amplitude electrical current is applied across the thorax (TEBT) or entire body (WBEBT). The resistance (impedance, Z0) to this electrical current varies between different tissues in the body, with the primary distribution being to the blood and extracellular fluid. This change in electrical current (ΔZ0) over time (dZ0/dt) corresponds to the level of SV, from which CO can be computed.

Electrical biosensing technology is divided into 2 broad categories: (1) bioimpedance (BI) which encompasses thoracic electrical velocimetry/electrical cardiometry, impedance cardiography as well as WBEBT and (2) bioreactance (BR).

### Electrical Velocimetry and Electrical Cardiometry

Electrical cardiometry (EC) is the method of non-invasive cardiac output technology that utilizes the model of thoracic electrical velocimetry (EV) to determine SV and CO (16). These are used by Aesculon and ICON, manufactured by Osypka Medical GmbH, Germany.

In EV, the change in impedance (ΔZ0) is due to the degree of erythrocyte alignment in the aorta throughout the cardiac cycle. During diastole, as the aortic blood flow ceases, erythrocytes are randomly orientated and interfere with electrical conduction. During systole as the left ventricle contracts, the erythrocytes are forced to align parallel to aortic flow and the electrical current in the aorta passes with less impedance, hence an increased conductivity. These pulsatile changes in volume and thus in impedance, in relation to the cardiac cycle (ΔZ0(t)), are used to calculate SV.

EV estimates SV by means of the following equation:


(1) $${\text{SV}}_{\text{TEB}}=\text{CP}{\text{.v}}_{\text{ft}}.\text{FT}$$

where SV_*TEB*_ is SV estimated by thoracic electrical bioimpedance, CP is the patient constant (in ml), v_*ft*_ is the mean blood velocity index (in s^–1^) during flow time (FT; measured in s). The EV model estimates SV based on the input of the patient’s body mass, an empiric means velocity index derived from a peak amplitude measurement assumed to be the peak aortic blood flow acceleration and a measurement of flow time.

### Impedance Cardiography

Impedance cardiography (ICG) and electrical cardiometry (EC) are similar as both rely on periodical volumetric changes in the aorta to determine SV and CO. However, ICG and EC differ in the model applied to determine impedance measurements, specifically as to how the change in impedance is calculated. In ICG the change in impedance (conductivity) (ΔZ(t)) is solely attributed to the volumetric expansion of the ascending aorta due to the increase of volume within the aorta or due to its wall motion. The index of peak velocity of the volumetric change is used in ICG as compared to the index of peak acceleration in EV. EV includes direction of flow whereas ICG does not. In EV, volume changes also incorporate the alignment of erythrocytes.

### Bioreactance

In Bioreactance (BR), another thoracic EBT method, it is assumed that blood flow changes are not only related to changes in impedance but also changes in capacitance (ability of biological tissue to store an electrical current) and inductance (biological tissue’s ability to store energy in a non-electrical form). BR therefore measures phase shift (φ) of an oscillating current as it traverses the thorax. Four pairs of sensors, one electrode acting as a high frequency generator and the other as a receiver, are placed on either side of the thorax. CO measurements are determined separately from each side of the body and the final CO is the average of the measurements. BR uses the following formula to estimate SV:


(2) $$\mathrm{SV}=C\times \mathrm{VET}\times d\varphi /\mathrm{dt}{}_{\max}$$

where C is a constant of proportionality, VET is ventricular ejection time, and dφ/dt_max_ is the peak rate of change of the phase shift (φ). BR is used by the Reliant and its newer version, Starling, manufactured by Baxter, United States.

Significant differences exist between BI and BR (Table 1).

**TABLE 1:** Comparison of technological differences between bioimpedance and bioreactance.

	Bioreactance	Bioimpedance
Assumptions regarding blood flow	Related to change in body’s resistance, capacitance, and inductance	Related to the change in body’s resistance only
Anatomical assumption regarding thorax	Thorax is an electrical circuit with resistor and capacitor	Fluid-filled cylinder or truncated cone
SV estimation	Calculated from phase shift, determined from resistance and capacitance [amplitude magnitude of impedance and phase direction of impedance (Z0)]	Calculated from estimated changes in resistance/impedance (Z0) – electrical current flow over time due to aortic blood flow changes
SV calculation	Peak rate of change of the phase shift (dφ/d*t*_max_) is proportional to the peak aortic flow from which SV is calculated (eq. 2)	Instantaneous rate of change in Z0 is related to aortic blood flow and SV is proportional to the maximal rate of change of Z0 (dZ0/d*t*_max_) and the ventricular ejection time (VET)
Electrode placement	Applied at the base of the neck (thoracic inlet) and costal margins (thoracic outlet) on both sides. Measurements are sensitive to electrode placement	2 pairs of dual-electrode sensors – placed on the left thorax and right neck Measurements stated not to be sensitive to placement
VET determination	VET is determined by BR (first and second zero crossing of the dφ/d*t* signal) and electrocardiographic signals (peak of the QRS complex)	VET is determined by the distance between QRS complexes

*Modified from Jakovljevic et al., (17) dZ0/dt, impedance change over time; VET, ventricular ejection time; Z0, impedance; dφ/dt_max_, peak rate of change od change of phase shift.*

The aim of this systematic review was to identify and analyze studies that describe the accuracy, precision, and trending ability of TEBT to non-invasively monitor cardiac output and/or stroke volume in newborn infants.

## Methods

A systematic search was performed on PubMed NCBI, SCOPUS, and EBSCOHost to identify English language studies published up until 30 November 2021. Search terms included: non-invasive cardiac output, thoracic impedance, bioreactance, whole body bioimpedance, electrical velocimetry, electrical cardiometry, impedance cardiometry, impedance cardiography, cardiac output, stroke volume, neonate, newborn, and infant.

Studies on human neonates, term or preterm of any gestational and postconceptional age, were eligible for inclusion. The search strategy did not explicitly exclude animal studies to ensure that studies reporting animal and human research would be identified. However, studies reporting pure animal data were excluded upon screening of the title and abstract. Studies describing accuracy, precision, agreement, or trending data for non-invasive cardiac output monitors, as compared to a standard reference technique were eligible for inclusion. Thoracic and whole body electrical biosensing technology, encompassing bioimpedance and bioreactance technologies, were eligible for inclusion. Any standard comparative technology [transthoracic echocardiography (TTE), thermodilution techniques, Fick principle derived methods, or cardiac MRI (cMRI)] to compare to the investigational technology were eligible for inclusion. Studies investigating cardiac output (CO), or stroke volume (SV) were eligible. Studies that did not compare an investigational technology to a reference technology were excluded.

Any study reporting outcome measures allowing validation of EBT technologies were included. All published and pre-print manuscripts pending publication were eligible for inclusion.

Studies were selected for inclusion by screening titles and abstracts against selection criteria by 2 independent reviewers (LVW and WPdB) and conflicts were resolved through discussion, arbitrated by a third reviewer (SG), as required. Full text articles of the included studies were reviewed by the same reviewers to confirm eligibility and perform data extraction.

Extracted data included study details (first author, year of publication and study population details), investigational technology device name, comparator technology type and outcome data. If outcome data were not specifically reported, but were calculatable from provided data, the missing outcome measures were calculated. Where median and interquartile range (IQR) were reported, the mean and SD were calculated (18). Where SEM was reported, SD was calculated as SD = SEM ×√n. Where studies presented both CO and SV data, only data on which percentage error (PE) was calculated were used. Neonatal TEBT vs. TTE comparison studies only included data for left ventricular CO and SV.

Outcome measures were defined as follows:

For agreement (accuracy and precision) (12):

1.
  (a) Bias CO or SV, mean difference (investigative technology – comparator).
  (b) Mean CO or SV ([investigative technology + comparator]/2).
  (c) Percentage error (1.96 × SD of bias/mean ×100%).
  (d) Limits of agreement (bias ± 1.96 × SD).
  (e) True precision (TP) of comparator correcting for the true precision of TTE’s precision of ±30%: TP = √(PE)^2^−(0.3)^2^.

For trending ability (19):

1. Concordance rate: number of sequential changes in CO (ΔCO) or SV (ΔSV) (data points in concordant quadrants/all data points) × 100%.
2. Mean angular bias: average angle between all polar data points and polar axis.
3. Radial limits of agreement (LOA): defined as the radial sector containing 95% of data points (mean angular bias ±1.96 × SD).
4. Angular concordance rate: calculated as the percentage of data points in the ±30° radial zone.

## Strategy for Data Synthesis

Data synthesis strategy was defined by the extracted data. Although included studies were quantitative in nature and data were descriptively summarized, data gathered in this review were considered to be too heterogenous to allow statistical pooling for meta-analysis. There was variable reporting of measured hemodynamic parameters (CO or SV), hemodynamic parameter measurement unit (weight indexed vs. non-weight indexed) as well as data reporting in a wide variety of clinical situations. Studies were thus grouped primarily according to hemodynamic parameter, type of measurement unit, and according to clinical variables and interventions.

Heterogeneity was visually assessed by tabulation of the primary author, year of publication, hemodynamic variable, measurement unit, investigational technology, study population (gestational age, postnatal age, and birth weight) and interventions. Data were analyzed in line with these factors.

Effect direction plots were utilized to visually synthesize the diverse outcome measures, providing a link between the data and the narrative (20). Size and direction of arrows were used to indicate sample size, bias direction, precision, and percentage error (above or below 30% benchmark). Results were presented for overall results as well as for sub-analyses.

This review was performed according to the synthesis without meta-analysis (SWiM) guideline (21).

## Results

**FIGURE 1:**
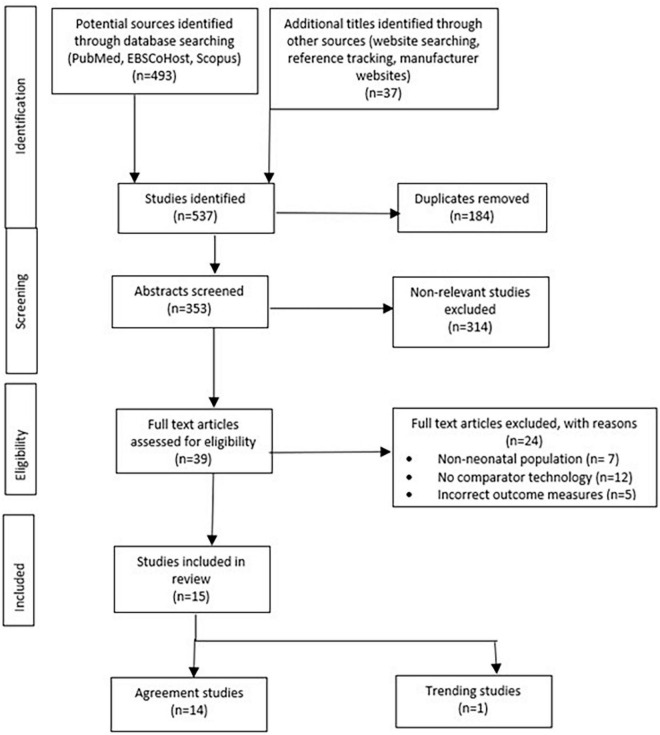
Flow chart of literature search strategy.

From an initial search, 295 studies were identified, which were assessed for eligibility. After full-text review, 15 studies were included, of which 14 were agreement studies (22–35) and 1 was a trending analysis study (36) (Figure 1 and Table 2).

**TABLE 2:** Study characteristics of included neonatal studies comparing TEBT and a reference technology.

References	Patient characteristics	Gestational age (weeks) (mean ± SD)	Birth weight (kg) (mean ± SD)	Postnatal age (days) [median (range)]	Investigative method (model used)	Reference method	Sample size [patients (measurements)]	Variable measured (unit)
**Agreement studies (accuracy and precision)**								
Tibballs (22)	Post cardiac surgery and RDS 100% IV Inotropes – NS	Prem and term	0.75–4.95	17 (0–121)	BI (NCCOM3)	TTE	26 (81)	CO (ml/kg/min)
Grollmuss (23)	TGA switch procedure 100% IV Inotropes: NS	NS	3.3 ± 0.5	10 (3–29)	BI (Aesculon)	TTE	24 (240)	SV (ml)
Noori (24)	40% PDA	39.2 ± 1.1	3.09 ± 0.33	4 (1–13)	BI (Aesculon)	TTE	20 (115)	CO (ml/min)
Weisz (25)	10% IV and 20% NIV	37 ± 6.6	2.72 ± 1.23	NS	BR (Reliant)	TTE	10 (97)	CO (ml/min) SV (ml)
Blohm (26)	4.9% IV 38.3% NIV 5% PDA	25–34	1.66 (0.84–2.40)	NS	BI (Aesculon)	TTE	26 (40)	
Grollmuss (27)	67% IV	31.7 ± 3.1	3.1 ± 1.61	15 (1–48)	BI (ICON)	TTE	28 (228)	SV (ml/kg)
Song (28)	50.4% IV 49.4% NIV 64% PDA 42.5% inotropes	27 ± 2.96	1.07 ± 0.78	0–1.5	BI (NS)	TTE	40 (109)	CO (ml/kg/min)
Weisz (29)	PDA ligation 88% IV 100% IV 52% inotropes	30.6 ± 2.51	0.7 ± 0.11	NS	BR (Reliant)	TTE	25 (78)	CO (ml/kg/min)
Torigoe (30)	29.6% IV 45.6% NIV 100% PDA	32 ± 2.9	1.63 ± 0.53	NS	BI (NS)	TTE	28 (81)	CO (ml/min)
Blohm (31)	4% PDA 42% PFO 21% PDA and PFO	Preterm and term	3.3 ± 2.51	1.9 (0.16–240)	BI (Aesculon)	TTE	99 (291)	CO (ml/min)
Boet (32)	31.6% IV 41.8% NIV 4% PDA	31 ± 3.2	1.11 ± 0.53	0–7	BI (NS)	TTE	79 (451)	CO (ml/min) SV (ml)
Hsu (33)	67% IV 35% NIV 100% PDA	27.2 ± 6.6	1.01 ± 1.00	6 (2–22)	BI (Aesculon)	TTE	36 (105)	CO (ml/kg/min)
Van Wyk (34)	70% NIV	31.3 ± 2.7	1.56 ± 0.41	0–3	BR (Reliant)	TTE	63 (754)	CO (ml/kg/min) SV (ml/kg)
Hassan (35)	8% HFO 46% SIMV 13% NIMV 25%CPAP 16% PDA	25.2 (22.3–31.6)	668 ± 157	24 (9–80)	BI (ICON)	TTE	38 (85)	CO (ml/min)
**Trending analysis studies**								
Van Wyk (36)	70% NIV	31.3 ± 2.7	1.56 ± 0.41	0–3	BR (Reliant)	TTE	63 (691)	CO (ml/kg/min) SV (ml/kg)

*BI, bioimpedance; BR, bioreactance; CO, cardiac output; ICON, index of contractility monitor; IV, invasive ventilation; NCCOM3, non-invasive computerized cardiac output monitor; NIV, non-invasive ventilation; NS, not specified; PDA, patent ductus arteriosus; PFO, patent foramen ovale; SD, standard deviation; TTE, transthoracic echocardiography.*

All studies were observational, prospective, method comparison studies. No study utilized whole body electrical biosensing technology (WBEBT), therefore only thoracic electrical biosensing technologies (TEBT) were included. The only reference method utilized in the included studies was transthoracic echocardiography (TTE). Investigational technologies comprised bioreactance (BR) (3 studies) and bioimpedance (11 studies) (Table 2). BI included: NCCOM3 (1 study), Aesculon/ICON (7 studies) and technology not specified in 3 studies. BR included NICOM^®^ Reliant (3 studies) (Table 2).

Most studies (*n* = 7) reported CO measurements only, 2 studies reported CO and SV, 2 studies reported SV only and 1 study reported TTE-VTI (velocity time integral) as well as TTE-MM (M-mode). Most studies (*n* = 7) reported weight-indexed measurements, 6 studies reported non-weight indexed measurements, and 1 study reported both (Table 2).

Thirteen studies reporting accuracy and precision outcome measures could be identified with only 1 neonatal study reporting trending data. Descriptive tables were ordered by hemodynamic parameter, weight-indexed or non-weight-indexed measurement and sample size.

The 14 eligible agreement studies involved a total number of 504 patients, encompassing 2668 paired measurements. Study sample sizes were generally small [average number of patients per study 38.7 (range 10–99)] with only 3 studies recruiting more than 50 patients. The average number of paired measurements per patient was 5.4 (range 1.5–11.9) with only 5 studies performing more than 5 paired measurements per patient (Table 2).

Studies varied widely in gestational ages, birth weight and chronological ages of enrolled neonates. Disease states, ventilation requirements and surgical intervention also varied widely between studies (Table 2), thereby illustrating the high heterogeneity between studies.

### Agreement Analysis

To determine the overall agreement (i.e., accuracy and precision) of TEBT vs. TTE, all studies were included in the analysis (Table 3). Most studies (8 out of 14) were large (>100) in sample size (number of measurements). An equal number of studies (7 of 14) showed that mean bias was positive or negative, indicating an overestimation and underestimation, respectively, of TTE measurements. The effect direction plots show a visual representation of sample sizes as well as the overestimation and underestimation of measurements (Table 4).

**TABLE 3:** Overall data regarding accuracy and precision of TEBT and reference technology.

Authors/Year	Unit of measurement	Number Patients (measurements)	Mean*	Bias** Mean ± SD	LOA (precision)	Overall PE
**Stroke Volume**						
Grollmuss (23)	ml	24 (240)	3.7	**0.28**	**±2.3**	29
Weisz (29)	ml	25 (78)	1.25	**−0.6** (39%)	±0.75	**58.0**
Blohm (31)	ml	99 (291)	5.2	0.7	±2.35	44.9
Boet (32)	ml	79 (451)	NS	1.1	±1.85	NS
**Cardiac Output**						
Tibballs (22)	ml/kg/min	26 (78)	239	0.23 ± 6.5	±13.50	5.3
Grollmuss (27)	ml/kg/min	28 (228)	256.4	8.9 ± 31.9	±62.7	24
Song (28)	ml/kg/min	40 (109)	209.5	−18.8 ± 67.7	±132.7	60.2
Hsu (33)	ml/kg/min	36 (105)	258	−5.3 ± 37.2	±72.9	28.2
Van Wyk (34)	ml/kg/min	63 (754)	124.4	−18.5	±87.6	71.6
Noori (24)	ml/min	20 (115)	536	−4	±233	43.6
Weisz (25)	ml/min	10 (97)	417	−153 ± 56	±152.5	48.3
Torigoe (30)	ml/min	28 (81)	314	6 ± 46.9	±66.5	21
Hassan (35)	ml/min	38 (85)	271	−126	±178.5	66
**VTI**						
Blohm (26)	m	26 (41)	NS	39%	NS	46.2

*Mean = (mean TEBT + mean TTE)/2; **Bias = TEBT – TTE. LOA, limits of agreement; NS, not stated; PE, percentage error; SD, standard deviation; TEBT, thoracic electrical biosensing technology; TTE, transthoracic echocardiography; VTI, velocity time integral. Bold values have been calculated from available data or determined from provided from graphs.

**TABLE 4:** Effect direction plots for TEBT studies: overall accuracy and sub-analyses of clinical conditions.

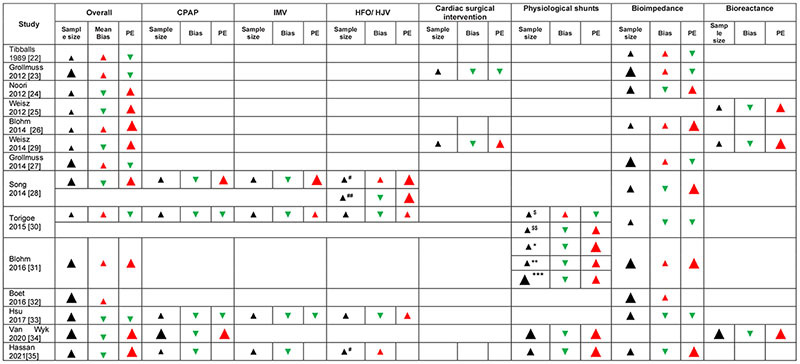

^#^HFO ^##^HFJ; *PDA only, **PFO and PDA, ***PFO only; ^$^PDA < 1.5 mm; ^$$^PDA ≥ 1.5 mm. CPAP, continuous positive airway pressure; HFO, high frequency oscillation; HFV, high frequency jet ventilation; IMV, intermittent mandatory ventilation; NS, not specified; PDA, patent ductus arteriosus; PFO, patent foramen ovale; PE, percentage error; VTI, velocity time integral. Legend: Sample size (based on number of measurements: large arrow – large sample size >100, medium arrow 50–100, and small arrow <50). Bias effect: Downward green arrow – TEBT underestimates reference method, upward red arrow – TEBT overestimates reference method; size of arrow indicates degree. Percentage error: downward green arrow PE < 30%, upward red medium arrow 30–50%, upward large red arrow >50.

Non-weight indexed SV mean bias was reported in 4 studies and varied between 0.6 to 1.1 ml. Limits of agreement (LOA) varied between ±0.75 and ±2.35 ml. Percentage error (PE), reported in only 3 studies, varied between 29 and 58% (Tables 3, 4).

CO mean bias measurement was reported in 9 studies and ranged between 8.9 to −18.5 ml/kg/min for weight indexed and 6 to −153 ml/min for non-weight-indexed CO. LOA varied between ±13.5–132.7 ml/kg/min and ±66.5–233 ml/min, respectively. PE ranged between 5.3 to 71.6%, with only 5 of the 14 included studies reporting a PE ≤ 30% (Tables 3, 4).

One study used VTI as hemodynamic reference parameter. The bias percentage was 39% and PE was 46%.

In order to determine whether there were differences in accuracy and precision when studies were performed with different ventilation modes, the 5 studies reporting outcome measures for various respiratory support methods were analyzed (Table 5). Five studies reported outcome measures for non-invasively ventilated (NIV) infants and 4 studies for invasively ventilated (IV) infants.

**TABLE 5:** Accuracy and precision in studies with different respiratory support modes.

Authors	Unit of measurement	n Measurements	Type of respiratory support	Mean*	Bias** Mean ± SD	LOA (precision)	PE
**Non-invasive ventilation**							
Song (28)	ml/kg/min	54	CPAP	209	−18.2 ± 63.7	±124.9	57
Hsu (33)	ml/kg/min	37	CPAP	258	−2.8 ± 20.8	±40.7	17.3
Van Wyk (34)	ml/kg/min	335	CPAP	124.4	−23.0 ± 45.8	±84.8	78.5
Torigoe (30)	ml/min	37	CPAP	314	3.6	±73.1	25.0
Hassan (35)	ml/min	21	CPAP	268	−178	NS	NS
		11	NIMV	291	−138	NS	NS
**Invasive ventilation**							
Song (28)	ml/kg/min	39	SIMV	209	−30.2 ± 73.8	±94.6	69.7
Torigoe (30)	ml/min	10	SIMV	314	−29.6	±97.8	31.7
Hsu (33)	ml/kg/min	44	IMV	258	−1.4 ± 36.0	±70.1	27.4
Hassan (35)	ml/min	39	SIMV/ACV	243	−115	NS	NS
Song (28)	ml/kg/min	8	HFOV	209.5	38.2 ± 91.4	±179.1	85.5
Torigoe (30)	ml/min	14	HFOV	314	−12.0	±105.9	33.2
Hsu (33)	ml/kg/min	24	HFOV	258	−16.2 ± 40.4	±79	37.8
Song (28)	ml/kg/min	8	HFJV	209	−10.9 ± 81.7	±160.1	76.4
Hassan (35)	ml/min	7	HFOV	177	−21.5	NS	NS

*Mean = (mean TEBT + mean TTE)/2; **Bias = TEBT – TTE. CPAP, continuous positive airway pressure; HFOV, high frequency oscillatory ventilation; HFJV, high frequency jet ventilation; ACV, assist control ventilation; LOA, limits of agreement; NS, not stated; PE, percentage error; SD, standard deviation; SIMV, synchronized intermittent mandatory ventilation; TEBT, thoracic electrical biosensing technology; TTE, transthoracic echocardiography. Bold values have been calculated from available data or determined from provided from graphs.

In the NIV group, most studies reported outcomes for CPAP whereas 1 study reported non-invasive intermittent ventilation outcome measures. Sample sizes were small (8–54 measurements) with one large study (>100 measurements). TEBT underestimated TTE in 4 out of 5 studies. Weight-indexed CO mean bias varied between −2.8 and −23.0 ml/kg/min with wide LOA (±40.7 to 124.9 ml/kg/min). Non-weight-indexed CO mean bias varied between 3.6 and −178 ml/min with LOA between ±70.1 to ±94.6 ml/kg/min and ±LOAs available. PE for NIV studies ranged between 17.3 to 78.5% with only 2 out of 5 NIV studies having a PE ≤ 30% (Table 5).

For the IV group, outcome measures were reported in 4 studies utilizing various intermittent mandatory ventilation (IMV) modes [synchronized IMV, assist control ventilation, 4 studies utilizing high frequency ventilation (HFV) and 1 study utilizing high frequency jet ventilation (HFJV)]. In all studies, sample sizes were small (<50 measurements). TEBT underestimated TTE in most studies (8 out of 9). For IMV modes, the weight-indexed CO mean bias was −1.4 to −30.2 ml/kg/min with wide LOA (±70.1 to ±94.6 ml/kg/min) and non-weight-indexed CO mean bias of −29.6 to −115 ml/min with only a single study providing LOA (± 97.8 ml/min). Three studies provided PE, ranging from 27.4 to 69.7%, with 3 studies providing PE with only 1 of the 3 of studies reporting a PE ≤ 30%. For HFV studies, CO mean bias was −16.2 to 38.2 ml/kg/min and −12 ml/min for weight-indexed and non-weight indexed measurements, respectively. LOA ranged between ±79 to ±179.1 ml/kg/min and only 1 non-weight-indexed study proving a LOA of ±105.9 ml/min. For HFV studies, PE ranged between 33.2–85.5% with no studies reporting a PE ≤ 30%. The single HFJV study reported a CO mean bias of −10.9 ml/kg/min and PE of 76.4% (Table 5).

In a cross comparison of all respiratory modes, mean bias, LOA and PE seemed to show an increase as the complexity of respiratory support increased (Table 6).

**TABLE 6:** Comparative outcome measures for type of ventilation for included studies.

		CPAP				IMV/SIMV				HFO/HFJV			
Authors	Unit of measurement	n	Mean bias	Precision	PE	*n*	Mean bias	Precision	PE	*n*	Mean bias	Precision	PE
Song (28)	ml/kg/min	54	−18.2	±124.9	57	39	−30.2	±94.6	69.7	8 (HFO)	38.2	±160.1	85.5
										8 (HFJ)	−10.9	±79	76.4
Hsu (33)	ml/kg/min	37	−2.8	±40.7	17.3	44	−1.4	±179.1	27.4	24 (HFO)	−16.2	±70.1	37.8
Torigoe (30)	ml/min	37	3.6	±73.1	25	10	−29.6	±97.8	31.7	14 (HFO)	−12	±105.9	33.2
Van Wyk (34)	ml/kg/min	335	−23	±84.8	78.0								
Hassan (35)	ml/min	21	−21.5			39	−115			7 (HFO)	−21.5		

**Bias = TEBT – TTE. CPAP, continuous positive airway pressure; SIMV, synchronized intermittent mandatory ventilation; HFOV, high frequency oscillatory ventilation; HFJV, high frequency jet ventilation; LOA, limits of agreement; PE, percentage error; TEBT, thoracic electrical biosensing technology; TTE, transthoracic echocardiography.

The effect direction plots (Table 4) show a visual summary of the sample sizes, bias and PE for all respiratory support studies.

Numerous other studies included patients on CPAP but did not specifically report outcome measures for that subset of data (23, 25, 26, 32).

For NIV, Van Wyk et al. (34) showed a significant difference in weight-indexed CO bias between CPAP and no respiratory support (78.0 vs. 74.4%, *p* = 0.0.26) but not for weight-indexed SV bias (*p* = 0.113). Blohm et al. (26) showed a significant effect of CPAP on the bias between TEBT and TTE-VTI (*p* = 0.022) but not TEBT and TTE-MM (m-mode).

Song et al. (28) showed no significant difference in PE between CPAP and SIMV (57 vs. 69.7%, *p* = 0.160) nor SIMV and HFV (69.7 and 85.5%, *p* = 0.729). Hsu et al. (33) showed minimal change in bias between CPAP and IMV but a very large increase in bias between IMV and HFV, with incremental increases in PE from CPAP to IMV to HFV. Song et al. (28) reported a worsening of weight-indexed CO bias between neonates on CPAP and SIMV (−18.2 ml/kg/min and −30.2 ml/kg/min, respectively). Increased complexity of respiratory support intervention caused an increase in PE. Hassan et al. (35) showed that HFV only had a significantly lower bias (*p* = 0.002) as compared to IMV and CPAP.

Other studies reported no effect of respiratory support mode on CO bias. Torigoe et al. (30) showed no effect of mechanical ventilation on bias (estimated mean bias of 60 ml/min for no respiratory support and SIMV and approximately 25 ml/min for nCPAP and HFV, *p* = 0.14). Grollmuss et al. (23) reported that method interchangeability was not affected by respiratory support mode although no data was provided.

To determine whether there were differences in accuracy and precision when cardiac lesions were present, studies reporting outcome measures for different congenital cardiac lesions (pathological and physiological) were analyzed. Six studies were included. Most studies reported small sample sizes with only 1 study consisting of more than 100 measurements (Table 5). Most studies (6 out of 8) showed that TEBT underestimated TTE (Table 7).

**TABLE 7:** Accuracy and precision in studies with cardiac shunts.

Authors/Year	Unit of measurement	*n* measurements	Cardiac defect	Mean*	Bias ** Mean ± SD	LOA (precision)	PE
**Cardiac surgical intervention**							
Grollmuss (23)	ml	240	TGA switch surgery	3.7	**0.27**	**±1.06**	29.0
Weisz (29)	ml	78	PDA ligation	1.25	**−0.6** (39%)	**±0.75**	**58.0**
**Physiological shunt**							
Blohm (31)	ml	12	PDA only	4.1	−0.8 ± **1.73**	±0.98	72.1
		63	PDA + PFO		−1.1 ± **1.58**	±1.09	56.3
		125	PFO only		−0.6 ± **1.11**	±0.59	40.2
Van Wyk (34)	ml/kg/min	304	PDA	124.4	−28.7 ± 43.7	±78.1	74.4
Torigoe (30)	ml/min	23	PDA ≥ 1.5 mm	317	5.5	±66.2	21.0
		58	PDA < 1.5 mm		−36.1	±119.5	38.6
Hassan (35)	ml/min	14	DFLPA > 30 cm/s	**295.5**	−167	203.8	69

*Mean = (TEBT + TTE)/2. **Bias = TEBT – TTE. Bold data indicates calculated data or data estimated from provided graphs. DFLPA, end diastolic flow in left pulmonary artery; LOA, limits of agreement; PDA, patent ductus arteriosus; PE, percentage error; PFO, patent foramen ovale; SD, standard deviation; TGA, transposition of great arteries.

Two studies reported outcome measures in cardiac surgical interventions, The TGA switch study (23) showed a non-weight-indexed SV mean bias of 0.27 ml and a PE of 29%. The PDA ligation study (29) reported a non-weight-indexed SV mean bias of −0.6 ml and a PE of 58% (Table 7).

In studies (*n* = 4) reporting outcome measures for patients with a PDA, SV and CO mean bias were reported for both weight- and non-weight indexed measurements. PE in the 4 studies varied between 21 to 74.4%. Hemodynamic significant PDA was defined in only 2 studies (30, 35) (Table 7).

In studies (*n* = 2) reporting outcome measures for patients with a PFO, only 1 study reported data for PFO only and one for PDA combined with PFO. Both studies showed a high PE (40.2 and 56.3%, respectively) (Table 7).

The effect direction plots (Table 4) shows a visual summary of the sample sizes, bias and PE for all studies reporting data for cardiac lesions.

Other studies included patients with a PDA but did not specifically report outcome measures for that subset of data (28, 32). Various studies reported associations between PDA and mean bias without reporting specific outcome measures. Noori et al. (24) reported no statistically significant difference (*p* = 0.800) in bias (12 vs. 2 ml/min) or precision (±296 vs. ±218 ml/min) between neonates with a hemodynamically significant (ductal diameter >2 mm) PDA as compared to those without. Van Wyk et al. (34) reported a significantly higher mean bias for infants with an open PDA compared to a closed PDA (−28.7 ml/kg/min vs. −12.5 ml/kg/min, *p* < 0.001). Blohm et al. (26) showed that PDA status showed a trend toward significance when TEBT was compared to TTE measured VTI mean bias (*p* = 0.077) but not when TEBT was compared to TTE m-mode measurements. Hassan et al. (35) showed a near doubling in mean CO bias between PDA and non-PDA measurements (−167 ml/min and −89 ml/min, respectively, *p* < 0.001).

To determine if accuracy and precision was related to the type of TEBT technology utilized, studies were analyzed according to whether bioimpedance (BI) (*n* = 11) or bioreactance (BR) (*n* = 3) was used (Table 8).

**TABLE 8:** Outcome measure for studies using bioimpedance or bioreactance technology.

Authors year	Unit of measurement	n Patients (measurements)	Specific technology	Mean*	Bias ** Mean ± SD	LOA (precision)	Overall PE
**Bioimpedance**							
Grollmuss (23)	ml	24 (240)	Aesculon	3.7	**0.28 ± 0.05**	**±2.3**	29
Blohm (31)	ml	99 (291)	Aesculon	5.2	0.7	±2.35	44.9
Boet (32)	ml	79 (451)	NS	NS	1.1	**±1.85**	NS
Hsu (33)	ml/kg/min	36 (105)	Aesculon	258	−5.3 ± 37.2	±72.9	28.2
Tibballs (22)	ml/kg/min	26 (78)	NCCOM3	239	0.23 ± 6.5	±13.50	5.3
Grollmuss (27)	ml/kg/min	28 (228)	ICON	256.4	8.9 ± 31.9	±62.7	24
Song (28)	ml/kg/min	40 (109)	NS	209.5	−18.8 ± 67.7	±132.7	60.2
Noori (24)	ml/min	20 (115)	Aesculon	536	−4	±233	43.6
Torigoe (30)	ml/min	28 (81)	NS	314	6 ± 46.9	±66.5	21
Blohm (26)	m^#^	26 (41)	Aesculon	NS	39%	NS	46.2
Hassan (35)	ml/min	38 (85)	ICON	271	−126	±178.5	66
**Bioreactance**							
Weisz (29)	ml	25 (78)	Reliant	1.25	−**0.6 ± 0.37** (39%)	±0.75	**58.0**
Van Wyk (34)	ml/kg/min	63 (754)	Reliant	124.4	−18.5	±87.6	71.6
Weisz (25)	ml/min	10 (97)	Reliant	417	−153 ± 56	±152.5	48.3

^#^TTE VTI was hemodynamic parameter measured by TTE. *Mean = (TEBT + TTE)/2. **Bias = TEBT – TTE. Bold data indicates calculated data or data estimated from provided graphs. LOA, limits of agreement; NS, not specified; PDA, patent ductus arteriosus; PE, percentage error; PFO, patent foramen ovale; SD, standard deviation; TGA, transposition of great arteries.

In studies utilizing BI, 5 out of the 11 studies showed overestimation and 6 out of 11 showed underestimation of TTE hemodynamic parameters. SV mean bias varied between 0.28 and 1.1 ml with LOA between ±1.85 and ±2.35 ml. CO mean bias varied between −18.8 and 0.23 ml/kg/min and −4 to 6 ml/min with LOA between ±13.5 to ±132.7 ml/kg/min and ±66.5 to ±233 ml/min for weight-indexed and non-weight-indexed CO bias, respectively. PE varied between 5.3–46.2%, with 5 out of 10 studies meeting the PE < 30% benchmark (1 study did not provide PE data) (Table 8).

In studies utilizing bioreactance, all studies showed an underestimation of TTE hemodynamic parameters (Table 8). PE ranged between 48.3–71.6% with all studies exceeding the PE < 30% benchmark.

### Trending Analysis

The single neonatal study assessing trending parameters utilized bioreactance (36). The study showed a poor concordance rate (77.2%), high mean angular bias (28.6°) with wide angular LOA and poor angular concordance (17.4%). Exclusion zone size affected trending parameters. PDA and CPAP showed no association with trending parameters, but CO level was significantly associated with trending parameters.

No other studies performed formal trending analysis. However, 2 studies showed changing agreement parameters over time. The TGA switch study (23) also showed varying bias and PE dependent on timing of measurements after surgery: 24, 35, and 28% within 0–36, 36–72 and after 72 h of surgery, respectively. The PDA ligation study (29) also showed an increase in bias over time: 7.9% (6–8 h post-ligation) and 9.7% (16–18 h post-ligation) as compared to measurements 1-h post-ligation.

## Discussion

In this review of TEBT technology in neonates, 14 studies were found describing the agreement between bioimpedance (*n* = 11) and bioreactance (*n* = 3) technology and transthoracic echocardiography (TTE). A total of 542 neonates were enrolled in these studies, comprising 2755 paired measurements. Only 1 study was found describing trending analysis, comparing bioreactance to TTE, which comprised 63 neonates and 691 paired measurements.

### Thoracic Electrical Biosensing Technology Accuracy and Precision

In the current review, the mean bias (difference between TEBT technology and the TTE reference) was small in many studies but limits of agreement were wide, indicating acceptable accuracy but a lack of precision. This was similar to 2 adult TEBT systematic reviews, where bias was determined to be small [−0.22 l/min (37) and 0.03 l/min (38)] with wide LOA [−2.43; 1.99 l/min (37) and −2.78; 2.84 l/min (38)]. A pediatric systematic review showed similar small bias [−0.02 to −0.01 l/min (38, 39) and 3.4 ml/kg/min (39) and wide LOA −1.22; 1.18 l/min (38) and ±75.17 ml/kg/min (39). However, in the most recent pediatric systematic review (39), only 5 neonatal studies were included.

Most studies providing PE data (8 out of 13) in this review did not meet the clinically accepted percentage error benchmark of less than 30%, thereby indicating that TEBT is not interchangeable with the reference technology, TTE. PE represents the LOA adjusted for the mean of both methods and therefore represents the random error between the two methods. It depicts the intrinsic variations in the assessed hemodynamic parameter that are not linked to true changes of that parameter (CO or SV), but rather to the environment and random precision error of the investigated or reference technology (38). The commonly accepted 30% arises from the original cardiac output method comparison studies using thermodilution as the reference technology, which has an inherent precision of 20% or less. Thus, if a new technology has a similar precision to thermodilution (i.e., ±20%), the combination will lead to a total error of ±28.3%, which is commonly rounded off to 30%. Therefore, if a new technology has a percentage error of <30%, the technology has a similar percentage error to the reference technology and is therefore an acceptable alternative (11). However, this PE only holds true when the reference technology has the same PE as thermodilution, i.e., 20%. The inherent percentage error for other technologies is often higher. TTE has been shown to have a much higher PE (39–53%) than thermodilution and cannot be considered as accurate as thermodilution. For this reason, it has been suggested that the PE threshold should be increased to 45% to conclude agreement between the two methods, to compensate for the variability of the reference method (40). If this argument were to be followed in this review, it would only increase the studies meeting the benchmark by 1, i.e., 6 out of 13 studies having a PE < 45%.

The current review showed significant heterogeneity amongst studies regarding measured hemodynamic parameter, unit of measurement, gestational and postnatal age of included neonates as well as different management strategies (respiratory support modes, inotropic support) as well as presence of physiological shunts or congenital cardiac disease. This is similar to the adult and pediatric systematic reviews showing high heterogenic indices [79.2–93% (37–39)].

Sub-analyses for respiratory support mode, presence of physiological shunts or cardiac disease/cardiac surgery as well as type of EBT technology, showed persistence of small bias, wide LOA, and high PE. Type of EBT technology assessment also showed variable effect of bias directionality: BI studies showed variable under- and overestimation whilst BR showed consistent underestimation of hemodynamic parameters. These differences may be due to sample sizes and population heterogeneity of the included studies, or may be due to the varying underlying mathematical models of the different technologies.

Most studies were performed in preterm infants, confirming the interest by clinicians to determine CO in this vulnerable population.

Percentage error differed between studies depending on respiratory support mode. In general, mean bias and PE increased with complexity and intensity of respiratory support mode, with invasive ventilation having higher PE than non-invasive ventilation methods. Most studies reported PE exceeding the benchmark, suggesting that respiratory support modes made TEBT less interchangeable with TTE. In most studies, the degree of respiratory support was not stated, i.e., mean airway pressure, which may affect bias, due to the amount of air between the sensors and aorta interfering with the sensors ability to measure cardiac outflow.

In studies reporting accuracy and precision for cardiac anomalies, accuracy was reasonable, but precision was poor. PE exceeded the benchmark in most studies. However, different definitions were used to define PDA (size and flow velocity in the pulmonary artery) and the size of PFO was not defined. Two studies were performed in neonates undergoing cardiac surgical intervention, increasing the heterogenicity of results.

Heteroscedasticity implies proportionality of bias, or variability of changes with the magnitude of measurement (57), which is a problem with healthcare method comparison studies (40). In TEBT method comparison studies, this implies increasing bias (difference between TEBT and reference method) with increasing CO or SV. Three studies reported heteroscedasticity in CO or SV bias. Boet et al. (32) showed that TEBT overestimated TTE when SV > 2 ml and CO > 0.4 l/min. Van Wyk et al. (34) showed an increasing bias when CO ≥ 150 ml/kg/min as compared to <150 ml/kg/min (*p* < 0.001). Hsu et al. (33) showed a statistically significant increase in bias (*p* = 0.001) but no statistically significant difference in PE when comparing a CO ≥ 280 ml/kg/min as compared to a CO < 280 ml/kg/min. This may be clinically relevant in larger neonates or neonates with high output cardiac failure with high CO or SV and requires further research.

The mean bias represents the systematic error between measurements, i.e., the mean constant difference. Although bias and LOA are used to statistically define accuracy and precision, there is no consensus regarding acceptable clinical cut-off values for these factors. What, therefore, represents an acceptable bias in neonatal studies requires further research.

### Thoracic Electrical Biosensing Technology Trending Ability

In trending analysis, the benchmark is a concordance rate of >92%, an angular bias <5°, angular limits of agreement ±30° and an angular concordance of >90% (19). The single trending study in this review did not meet any of these criteria. Two other agreement studies with measurements over time (23, 29), showed a temporal increase in bias and PE. TEBT is therefore not able to accurately track CO, as compared to TTE.

In trending studies, exclusion zones are required. In the single published neonatal study (36), the choice of exclusion zones significantly influenced trending data results. It is unknown what the exclusion zones should be in neonatology and requires further research.

In method comparison studies, the aim is to determine whether a new technology’s accuracy, precision and trending ability is similar to that of a gold reference technology and can therefore be used interchangeably. However, even the most accurate technologies (thermodilution) are known to have a degree of error. However, the reference technology in all these studies was TTE, which is known to be a relatively inaccurate reference technology (11, 42). Therefore, it cannot be considered an accurate reference method. However, the lack of other reference technologies attests to the difficulties of invasive testing for CO or SV in sick and small neonates and the difficulties in performing cMRI studies. In many of the included method comparison studies, a variety of TTE methods were used to measure SV or CO – aorta measurements at the annulus (24, 32, 33, 35), valve hinge points (25, 26, 30, 34), and Darmon’s triangular technique (23, 27) or were not specified (28, 29). This inconsistency in standardization of TTE measurements may also contribute to the range of agreement and trending data found in this review.

There also remains a question as to the what the impedance signal (Z0) reflects. The classic assumption that the impedance signal (ΔZ0) originates from the contractile changes of the aorta and/or the alignment of red blood cells during systole and diastole has been questioned (42). Mathematical models have not been able to confirm the origin of the Z0 signal and numerous recommendations have been made to improve these models for future research (43). The impact of these assumptions on the integrated measurement of left ventricular output, right ventricular output, in the presence of PDA and patent foramen ovale in neonates also needs to be considered carefully.

Various United States health insurers have stated that bioimpedance “continues to be reasonable and necessary” in various adult cardiac disease processes (44), despite an earlier finding by the National Institute for Health Research of inadequate evidence to support its use (45). Various concerns regarding the accuracy of these non-invasive cardiac output monitors have been raised in adult and pediatric medicine (37, 38, 46). Despite this, these monitors have been used in neonatology in research and clinical environments for diverse scenarios: monitoring transition at birth (47, 48), cardiac adaptation after birth (49), patent ductus arteriosus (PDA)diagnosis (50), PDA ligation (29), PDA medical therapy (51), monitoring congenital heart disease (52, 53), managing neonatal hemodynamic shock (54) and sepsis (55), and to predict clinical outcomes (56).

Several technological and physiological aspects have to be met prior to routine use of non-invasive cardiac output monitors in the clinical environment: (1) validation against gold reference standards, (2) accuracy along the entire spectrum of gestational age and birth weight, (3) ability to provide continuous measurements in absolute numbers, (3) be reliable, practical and non-invasive, (4) easy to apply, (5) inexpensive for widespread use, (6) feasible, (7) useful in neonates with extra- and intra-cardiac shunts as well as congenital cardiac disease and (8) continuously recordable alongside other physiological monitors (1). Although many of the technical usability aspects (continuous measurements, ease of use, non-invasive, easy to apply, recordable alongside other physiological monitors) have been proven in various studies, this review suggests more research is required regarding the accuracy in different gestational ages, cardiac shunts and congenital heart disease and the technology should be validated against a true reference method.

## Conclusion

Thoracic electrical biosensing technology, irrespective of the type of technology, has a poor interchangeability with TTE in newborn infants for left ventricular cardiac output/stroke volume. High heterogeneity of patients and interventions in the neonatal population made direct comparisons of studies difficult. TTE, as a comparator in this review, however, cannot be considered an ideal reference method and studies evaluating TEBT against an accurate reference method are required. TEBT should be used with caution in the neonatal population for monitoring and determining therapeutic interventions.

## Data Availability Statement

The original contributions presented in the study are included in the article/supplementary material, further inquiries can be directed to the corresponding authors.

## Author Contributions

LV, SG, and W-PB contributed to conception and design of the study, performed literature search, and extracted data for analysis. LV wrote the first draft of the article. All authors contributed to manuscript revision, read and approved the submitted version.

## Conflict of Interest

W-PB declares that his research group previously received unrestricted research grants from, or equipment had been put at their disposal by the following companies: Transonic Systems Inc., Ithaca, NY, United States; PULSION Medical Systems, Feldkirchen, Germany; Osypka Medical, Berlin, Germany/San Diego, CA, United States; Cheetah Medical, Inc., Maidenhead, United Kingdom, and NI Medical, Petah Tikva, Israel. SG declares that his research and hemodynamic interest group had previously received support from Osypka Medical, Berlin, Germany/San Diego, CA, United States, as sponsors of NeoCard-UK conference and Q-NES symposium for educational purposes. The remaining authors declare that the research was conducted in the absence of any commercial or financial relationships that could be construed as a potential conflict of interest.

## Publisher’s Note

All claims expressed in this article are solely those of the authors and do not necessarily represent those of their affiliated organizations, or those of the publisher, the editors and the reviewers. Any product that may be evaluated in this article, or claim that may be made by its manufacturer, is not guaranteed or endorsed by the publisher.

## Abbreviations

BI: bioimpedance
BR: bioreactance
BP: blood pressure
CPAP: continuous positive pressure ventilation
CO: cardiac output
cMRI: cardiac MRI
EBT: electrical biosensing technology
EC: electrical cardiometry
EV: electrical velocimetry
HFV: high frequency ventilation
HR: heart rate
ICG: impedance cardiography
IV: invasive ventilation
LOA: limits of agreement
NICU: neonatal intensive care unit
NIV: non-invasive ventilation
PDA: patent ductus arteriosus
PE: percentage error
PFO: patent foramen ovale
SV: stroke volume
TEBT: thoracic electrical biosensing technology
TD: thermodilution
TTE: transthoracic echocardiography
WBEBT: whole body electrical biosensing technology
VTI: velocity time integral
Z0: impedance.
